# Ischemic insular damage and stress ulcer in patients of acute ischemic stroke

**DOI:** 10.1002/brb3.3529

**Published:** 2024-05-15

**Authors:** Peng Ding, Guojuan Chen, Yuling Yang, Tong Zhang, Wenxia Li, Liqin Yang, Xueqing Liu, Delin Yu, Wei Yue

**Affiliations:** ^1^ Department of Neurology, Clinical College of Neurology, Neurosurgery, and Neurorehabilitation Tianjin Medical University, Tianjin Huanhu Hospital Tianjin China; ^2^ Department of Neurology Tangshan Gongren Hospital Tangshan China; ^3^ Department of Ultrasonic Tianjin Huanhu Hospital Tianjin China; ^4^ College of Traditional Chinese Medicine North China University of Science and Technology Tangshan China

**Keywords:** acid‐suppressive drug therapy, acute ischemic stroke, insular damage, stress ulcers

## Abstract

**Background and aims:**

Stress ulcer (SU) is a common complication in patients with acute ischemic stroke. The relationship of infarction location and the incidence of SU was unclear. Herein, we aim to investigate the association between ischemic insular damage and the development of SU.

**Methods:**

Data were retrieved from the SPARK study (Effect of Cardiac Function on Short‐Term Functional Prognosis in Patients with Acute Ischemic Stroke). We included the patients who had experienced an ischemic stroke within 7 days. The diagnosis of SU was based on clinical manifestations, including hematemesis, bloody nasogastric tube aspirate, or hematochezia. Evaluation of ischemic insular damage was conducted through magnetic resonance imaging. Cyclo‐oxygenase regression analysis and Kaplan–Meier survival curves were used to assess the relationship between ischemic insular damage and the occurrence of SU.

**Results:**

Among the 1357 patients analyzed, 110 (8.1%) developed SUs during hospitalization, with 69 (6.7%) experiencing infarctions in the anterior circulation. After adjusting for potential confounders, patients with ischemic insular damage exhibited a 2.16‐fold higher risk of developing SUs compared to those without insular damage (*p* = .0206). Notably, among patients with infarctions in the anterior circulation, those with insular damage had a 2.21‐fold increased risk of SUs (*p* = .0387). Moreover, right insular damage was associated with a higher risk of SUs compared to left insular damage or no insular damage (*p* for trend = .0117). Kaplan–Meier curves demonstrated early separation among groups, persisting throughout the follow‐up period (all *p* < .0001).

**Conclusions:**

This study identified a significant independent correlation between ischemic insular damage, particularly on the right side, and the development of SU during hospitalization, indicating the need to consider prophylactic acid‐suppressive treatment for patients with ischemic insular damage.

## Graphical abstract

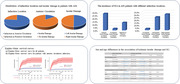

## Limitation

There were several limitations that should be addressed. First, it is important to acknowledge that some patients had been undergoing long‐term secondary prophylaxis with antiplatelet drugs, such as aspirin and clopidogrel, prior to their admission for cardiovascular or cerebrovascular conditions, and the use of such medications may have had an impact on the development of SUs. However, related data were not collected and may have influenced our findings to a certain extent. Additionally, it is worth noting that appropriate enteral nutrition is recognized as an effective strategy for preventing bleeding from SUs in critically ill patients, which can assist in buffering gastric acid, stimulating prostaglandin production, and improving regional mucosal perfusion (Braga et al., 2001; Ephgrave et al., 1990; Kazamias et al., 1998). However, in our study, we did not investigate the use of enteral nutrition or include it as an observed variable.

## 1 BACKGROUND

Stress ulcers (SUs) are acute mucosal erosions and ulcerations in the gastrointestinal tract, typically manifesting in multiple locations, most notably in the fundic parietal cell‐bearing area of the stomach and occasionally in the antrum and duodenum. SUs tend to develop in response to various stress‐inducing conditions, including critical illness, severe trauma, and serious mental disorders. Acute ischemic stroke (AIS) is a condition characterized by a high mortality rate and a risk of SUs during hospitalization (GBD 2016 Stroke Collaborators, 2019). The presence of SUs in AIS patients during hospital stays is often associated with an unfavorable prognosis, including the increased likelihood of complications, such as perforation, hemorrhagic shock, and even fatality. A significant portion of AIS patients receive aspirin (an antiplatelet medication) as part of their treatment. However, aspirin inhibits cyclo‐oxygenase‐1 (COX‐1) and can lead to gastric mucosal damage. Additionally, patients with cardioembolic strokes often require anticoagulants to prevent the formation of emboli, which can further complicate the management of gastric mucosal bleeding associated with SUs in AIS patients. Therefore, it is important to carefully consider prophylactic measures for SUs in AIS patients to mitigate these risks (Alhazzani et al., 2018).

The reduction in gastric mucosal blood flow and the occurrence of duodenogastric reflux are recognized as direct factors influencing the development of SUs in patients with AIS (Guilbert et al., 1969). It has been shown that specific regions of the brain exert significant influence over gastric mucosal blood flow. Notably, the insula, owing to its extensive neural connections, assumes a critical role in regulating autonomic nervous activity. Thus, patients with brain infarcts, particularly those involving the insular region, may face an elevated risk of developing SUs due to disruptions in neural control, thereby urging the need for more investigations into this phenomenon to reduce its occurrence and improve treatment outcomes. Furthermore, previous research findings suggest that the presence of a sympathetic effect site within the insular cortex of animal stroke models may also contribute to an increased incidence of SUs.

This study aims to identify robust predictors of SUs in patients with AIS to optimize the clinical use of acid suppression therapy, considering that accurately categorizing high‐risk patients may allow clinicians to provide targeted preventive treatment while avoiding unnecessary exposure to potential side effects of acid suppression therapy in low‐risk individuals.

## 2 MATERIALS AND METHODS

### 2.1 Study design and participants

This study enrolled patients diagnosed with AIS according to World Health Organization criteria at the Tianjin Huanhu Hospital Stroke Center between January 19, 2023, and March 20, 2023. The diagnosis was confirmed by specialist neurologists using computed tomography (CT) or magnetic resonance imaging (MRI) (Aho et al., 1980). The study inclusion criteria comprised individuals aged 18 and above with assessable neurological deficits (i.e., speech impairment, motor function impairment, cognitive impairment, gaze impairment, visual field deficits, or visual neglect) appearing within 7 days of hospitalization. Ethical approval for the study was obtained from the Committee for Medical Research Ethics at Tianjin Huanhu Hospital and the Tianjin Health Bureau, and informed consent was obtained from all participants or their legal guardians.

### 2.2 Data collection

Upon admission to the hospital, we collected demographic information from the patients, including their name, age, and gender. A comprehensive medical history was obtained, including details on hypertension (history of hypertension or use of antihypertensive medication), diabetes (a group of common endocrine diseases characterized by sustained high blood sugar levels), coronary heart disease (a type of heart disease where the arteries of the heart cannot deliver enough oxygen‐rich blood to the heart), and atrial fibrillation (an irregular heart rhythm that originates in the upper chambers of the heart). Additionally, we recorded the patients’ current smoking status and alcohol consumption. A neurologist conducted a thorough physical examination to assess the patients’ systolic and diastolic blood pressure, heart rate, and NIHSS score. The evaluation of three specific aspects, namely, TOAST classification, insular damage, and massive cerebral infarction, was performed using various diagnostic methods and imaging examinations, including CT, MRI, and magnetic resonance angiography. The definition of massive cerebral infarction included the following criteria: infarct lesions involving more than one third of the cerebral hemisphere within the supply area of the middle cerebral artery, as observed in CT or MRI imaging. Additionally, for infarct lesions in the cerebellum larger than 3 cm in size, as detected in CT or MRI imaging, the neurologist carefully assessed the location of cerebral infarct foci, documenting whether the infarction involved the insular cortex and specifying the hemisphere (left or right) for further analysis. Furthermore, the localization of the infarction within the anterior or posterior circulation was also recorded. In cases where infarctions affected both circulations, a small proportion of patients were categorized into the anterior circulation group. Patients were further classified into two groups based on the presence or absence of insular damage: the Insular Damage Group and the No Insular Damage Group. Additionally, during the patients’ hospital stay, we also documented the medications administered, and a specialized neurologist assessed whether each patient developed SU. Based on their SU status, the patients were categorized into two groups: the SU group and the non‐SU group.

### 2.3 Assessment of insular damage

Ischemic insular damage was evaluated using MRI, irrespective of the size of the infarct lesion. The assessment of ischemic insular damage was performed by neuroimaging specialists who examined the diffusion‐weighted images and were blinded to the patients’ clinical characteristics to ensure an unbiased evaluation.

### 2.4 Definition of SU

In this study, SUs in AIS patients were diagnosed through a combination of laboratory tests and clinical symptoms. Patients were classified as having concurrent SUs if they met either of two criteria: first, the presence of SUs with overt gastrointestinal bleeding, characterized by symptoms, such as hematemesis, the presence of bloody nasogastric tube aspirate, or melena (Saeed et al., 2022); and second, SUs with clinically significant gastrointestinal bleeding, defined as upper gastrointestinal bleeding accompanied by significant hemodynamic changes not attributable to other causes, including cases where bleeding was observed during upper gastrointestinal endoscopy or necessitated surgical intervention for management (Ye et al., 2020).

### 2.5 Statistical analysis

In our epidemiological survey, we divided our research population into two main groups: “anterior circulation infarction” and “posterior circulation infarction.” Within the anterior circulation infarction group, we further categorized patients into two subgroups: the “Insular Damage Group” and the “No Insular Damage Group” for a more detailed baseline description. We used COX regression analysis in all study populations, employing three distinct models. Covariates in the regression analyses were chosen based on their significance in relation to the outcome of interest and clinical relevance. Model 1 was unadjusted, whereas Model 2 incorporated adjustments for age, gender, and the admission NIHSS score. Model 3 extended Model 2 by including additional covariates, such as age, gender, admission heart rate, systolic blood pressure, admission NIHSS score, hypertension, diabetes, smoking, drinking, massive cerebral infarction, intravenous thrombolysis, endovascular treatment, anticoagulant treatment, aspirin treatment, and TOAST subtype, which allowed us to thoroughly assess the impact of ischemic insular damage on SU occurrence.

To enhance the reliability of our conclusions, we conducted an additional sensitivity analysis specific to the Anterior Circulation Infarction group, mirroring our original models. In this process, we further divided the Anterior Circulation Infarction group into three subgroups: the “No Insular Damage Group,” the “Left Insular Damage Group,” and the “Right Insular Damage Group.” We assessed normality using the Kolmogorov–Smirnov test and by examining histograms and Q–Q plots. For categorical variables, we used the *χ* ^2^ test, whereas the Kruskal–Wallis test was applied to NIHSS scores, and the one‐way ANOVA variance tests were employed for other normally distributed continuous variables. Additional baseline characteristics of anterior circulation infarction are shown in Table S1. Following the baseline analysis, COX regression analysis was used to compare the “No Insular Damage Group,” the “Left Insular Damage Group,” and the “Right Insular Damage Group.” The results were quantified with individual *p*‐values, and an overall *p*‐value for trend was calculated to identify any potential differential impact of left or right insular damage on the prevalence of SUs.

Kaplan–Meier survival analyses were separately conducted for the entire study population and the anterior circulation infarction group to assess the survival probabilities and event occurrence rates over time for both groups.

Statistical significance was determined for two‐sided *p* values <.05. All data analysis was performed using the SPSS software (version 26.0).

## 3 RESULTS

### 3.1 Baseline characteristics

A total of 1357 patients were included in this study, comprising 1037 individuals in the anterior circulation infarction group and 320 in the posterior circulation infarction group. The baseline characteristics of the study population are summarized in Table 1. Among the 1357 patients, 110 (8.1%) developed SUs following AIS during their hospital stay. When compared to patients without insular damage, those with insular damage exhibited more severe conditions, a higher prevalence of coronary heart disease, atrial fibrillation, large‐artery atherosclerosis subtype, cardioembolic subtype, massive cerebral infarction, endovascular treatment, anticoagulant treatment, and a higher incidence of SUs. Furthermore, notable differences were observed among the “No Insular Damage Group,” “Left Insular Damage Group,” and “Right Insular Damage Group” in terms of age, admission NIHSS score, admission heart rate, coronary heart disease, atrial fibrillation, stroke subtype according to TOAST classification, massive cerebral infarction, endovascular treatment, and the incidence of SU (Table S1).

**TABLE 1:** Baseline characteristics of the study population (*n* = 1357).

	Infarction in anterior circulation (*n* = 1037)		
Variables	Insular damage (*n* = 159)	No insular damage (*n* = 878)	Infarction in posterior circulation (*n* = 320)
Stress ulcer, *n*	44 (27.7%)	25 (2.8%)	41 (12.8%)
Men, *n*	118 (74.2%)	642 (73.1%)	242 (75.6%)
Age, mean (SD), years	67.25 (± 12.69)	63.33 (± 11.50)	62.59 (± 11.12)
Admission NIHSS score, median (IQR)	12 (8–17)	5 (2–8)	3 (1–6)
Admission heart rate, mean (SD), bmp	76.58 (± 17.29)	72.34 (± 13.16)	73.01 (14.12)
Systolic blood pressure, mean (SD), mmHg	156.63 (± 26.13)	149.58 (± 23.58)	151.45 (± 22.81)
Diastolic blood pressure, mean (SD), mmHg	87.80 (± 14.56)	86.86 (± 14.27)	88.52 (± 14.21)
Hypertension, *n*	113 (71.1%)	671 (76.4%)	254 (79.4%)
Diabetes, *n*	53 (33.3%)	314 (35.8%)	144 (45.0%)
Coronary heart disease, *n*	43 (27.0%)	127 (14.5%)	53 (16.6%)
Atrial fibrillation, *n*	29 (18.2%)	34 (3.9%)	18 (5.6%)
Living habits, *n*			
Current smoking	90 (56.6%)	472 (53.8%)	170 (53.1%)
Current drinking	55 (34.6%)	261 (29.7%)	104 (32.5%)
Stroke subtype by TOAST, *n*			
Large‐artery atherosclerosis	109 (68.6%)	399 (45.4%)	175 (54.7%)
Cardioembolic	35 (22.0%)	44 (5.0%)	22 (6.9%)
Small‐artery occlusion	2 (1.3%)	232 (26.4%)	69 (21.6%)
Other etiology	1 (0.6%)	18 (2.1%)	6 (1.9%)
Undetermined	12 (7.5%)	185 (21.1%)	48 (15.0%)
Massive cerebral infarction, *n*	69 (43.4%)	6 (0.7%)	30 (9.4%)
Intravenous thrombolysis, *n*	10 (6.3%)	57 (6.5%)	27 (8.4%)
Endovascular treatment, *n*	12 (7.5%)	3 (0.3%)	4 (1.3%)
Anticoagulant treatment, *n*	43 (27.0%)	157 (17.9%)	83 (25.9%)
Aspirin treatment, *n*	150 (94.3%)	842 (95.9%)	301 (94.1%)

Abbreviations: IQR, interquartile range; NIHSS, National Institutes of Health Stroke Scale; SD, standard deviation; TOAST, Trial of ORG 10172 in Acute Stroke Treatment.

### 3.2 Ischemic insular damage and SU

The results of the COX regression analysis examining the relationship between ischemic insular damage and SUs are presented in Table 2.

**TABLE 2:** The cyclo‐oxygenase (COX) regression analysis for the association of ischemic insular damage with stress ulcer.

	Ischemic insular damage		No ischemic insular damage		*p*‐Value
	HR	95%CI	HR	95%CI	
In the full population (*n* = 1357)					
Model 1	5.39	3.68–7.89	.19	.13–.27	<.0001
Model 2	4.97	3.37–7.33	.52	.33–.81	<.0001
Model 3	2.16	1.13–4.14	.46	.24–.89	.0206
In patients with infarction in anterior circulation (*n* = 1037)					
Model 1	10.91	6.68–17.84	.09	.06–.15	<.0001
Model 2	3.89	2.18–6.97	.26	.14–.46	<.0001
Model 3	2.21	1.04–4.69	.45	.21–.96	.0387

*Note*: Model 1, unadjusted; Model 2, adjusted by age, gender, and admission NIHSS score; Model 3, adjusted by age, gender, admission heart rate, systolic blood pressure, admission NIHSS score, hypertension, diabetes, smoking, drinking, massive cerebral infarction, intravenous thrombolysis, endovascular treatment, anticoagulant treatment, aspirin treatment, and TOAST subtype.

Abbreviations: CI, confidence interval; HR, hazard ratio; NIHSS, National Institutes of Health Stroke Scale; TOAST, Trial of ORG 10172 in Acute Stroke Treatment.

**FIGURE 1:**
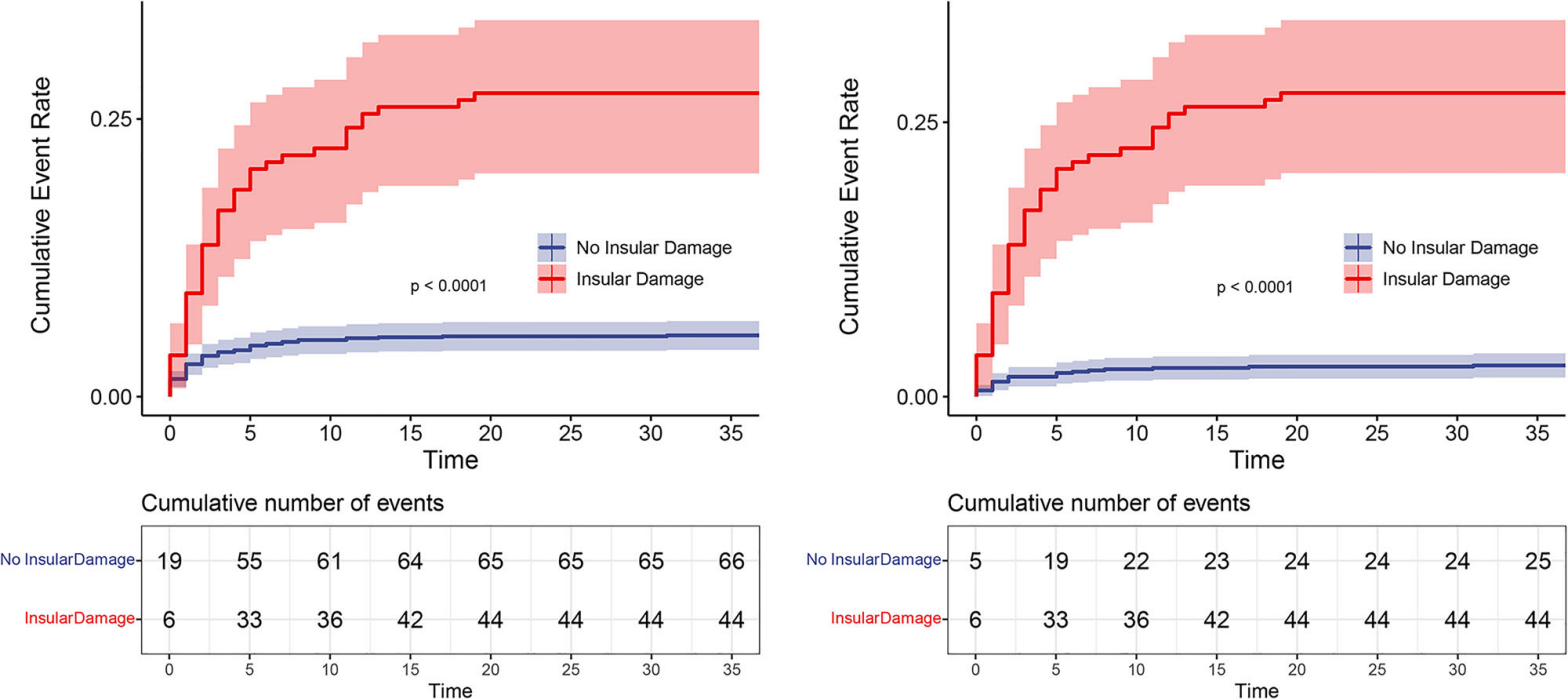
Kaplan–Meier survival curves: (a) Kaplan–Meier survival curve for all patients; (b) Kaplan–Meier survival curve for patients with anterior circulation infarction.

In the full population, when unadjusted in Model 1, ischemic insular damage exhibited a significant association with SU, with a hazard ratio (HR) of 5.39 (95% CI: 3.68–7.89, *p* < .0001). After adjusting for age, gender, and admission NIHSS score in Model 2, the association between ischemic insular damage and SU remained significant, with an HR of 4.97 (95% CI: 3.37–7.33, *p* < .0001). Further adjustments for age, gender, admission heart rate, systolic blood pressure, admission NIHSS score, hypertension, diabetes, smoking, drinking, massive cerebral infarction, intravenous thrombolysis, endovascular treatment, anticoagulant treatment, aspirin treatment, and TOAST subtype (Model 3) revealed that ischemic insular damage retained a significant association with SU, but with a slightly reduced HR of 2.16 (95% CI: 1.13–4.14, *p* = .0206). The Kaplan–Meier survival curves provided additional insights into the study's outcomes, showing a highly significant difference in survival probabilities (*p* < .0001) (Figure 1).

A sensitivity analysis was conducted in the subset of patients with anterior circulation infarction (*n* = 1037) using the same modeling approach as in the full population, and the results were similar to the findings from the overall analysis. In Model 1, the unadjusted analysis revealed a robust and statistically significant association between ischemic insular damage and SUs, with an HR of 10.91 (95% CI: 6.68–17.84, *p* < .0001). After adjusting for age, gender, and admission NIHSS score in Model 2, the association remained significant, with an HR of 3.89 (95% CI: 2.18–6.97, *p* < .0001). Similarly, in Model 3, which included additional adjustments using the same set of covariates as in the full population, the association remained statistically significant. In addition, the HR for ischemic insular damage and SUs was 2.21 (95% CI: 1.04–4.69, *p* = .0387). Further, the Kaplan–Meier survival curve illustrated a significant difference in survival probabilities among different groups (*p* < .0001) (Figure 1).

### 3.3 Left‐side and right‐side insular damages with SUs

Next, we further examined the influence of left‐side and right‐side insular damages on SU (Table 3). In Model 1, the model without any adjustments, the HR for left insular damage was 9.65 (95% CI: 5.36–17.39) and for right insular damage, it was 12.25 (95% CI: 6.99–21.45), with both comparisons displaying highly significant *p*‐values (< .0001). Model 2, which included adjustments for age, gender, and admission NIHSS score, yielded lower HRs for left insular damage (3.03, 95% CI: 1.53–6.00) and right insular damage (4.76, 95% CI: 2.55–8.88) (both *p* < .0001). In Model 3, the HRs were 1.78 (95% CI:.74–4.06) for left insular damage and 2.71 (95% CI: 1.23–6.04) for right insular damage (*p* = .1677 and.0138, respectively). Notably, there was a discernible trend indicating that patients with right insular damage had a higher risk of developing SU compared to patients with no insular damage and those with left insular damage (*p* for trend <.0001 in Model 1, <.0001 in Model 2, and.0117 in Model 3) (Table 3).

**TABLE 3:** The cyclo‐oxygenase (COX) regression analysis of left‐side and right‐side insular damages with stress ulcers (*n* = 1037).

	No insular damage	Left insular damage	Right insular damage	*p* for trend
	HR (95%CI)	HR (95%CI)	HR (95%CI)	
Model 1	1.00 (Reference)	9.65 (5.36–17.39)	12.25 (6.99–21.45)	<.0001
*p* Value		<.0001	<.0001	
Model 2	1.00 (Reference)	3.03 (1.53–6.00)	4.76 (2.55–8.88)	<.0001
*p* Value		.0015	<.0001	
Model 3	1.00 (Reference)	1.78 (.74–4.06)	2.71 (1.23–6.04)	.0117
*p* Value		.1677	.0138	

*Note*: Model 1, unadjusted; Model 2, adjusted by age, gender, and admission NIHSS score; Model 3, adjusted by age, gender, admission heart rate, systolic blood pressure, admission NIHSS score, hypertension, diabetes, smoking, drinking, massive cerebral infarction, intravenous thrombolysis, endovascular treatment, anticoagulant treatment, aspirin treatment, and TOAST subtype.

Abbreviations: CI, confidence interval; HR, hazard ratio; NIHSS, National Institutes of Health Stroke Scale; TOAST, Trial of ORG 10172 in Acute Stroke Treatment.

### 3.4 Sex and age differences in the association of ischemic insular damage and SU

**FIGURE 2:**
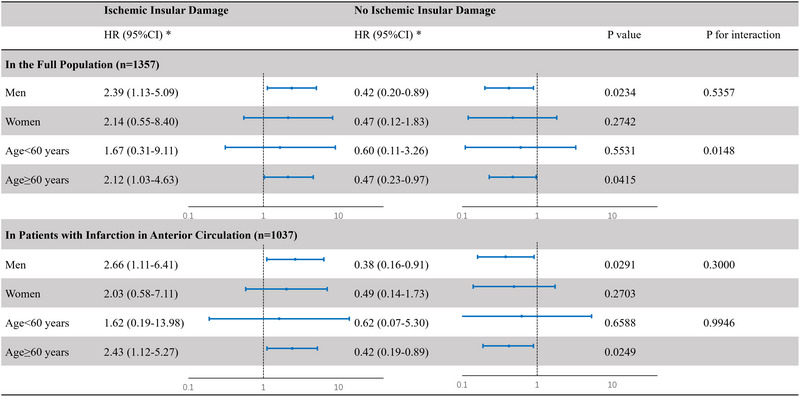
**Stratification analysis by age and sex**: Model 1, unadjusted; Model 2, adjusted by age, gender, and admission National Institutes of Health Stroke Scale (NIHSS) score; Model 3, adjusted by age, gender, admission heart rate, systolic blood pressure, admission NIHSS score, hypertension, diabetes, smoking, drinking, massive cerebral infarction, intravenous thrombolysis, endovascular treatment, anticoagulant treatment, aspirin treatment, and Trial of ORG 10172 in Acute Stroke Treatment (TOAST) subtype. CI, confidence interval; HR, hazard ratio.

The stratification analysis by age and sex is shown in Figure 2. In the full population, there was no significant interaction between ischemic insular damage and sex (*p* for interaction = .5357). A significant interaction was found between ischemic insular damage and age (*p* for interaction = .0148). Ischemic insular damage was associated with increased risk for SU in the patients of ≥60 years (HR: 2.12; 95%CI: 1.03–4.63), but not in the patients of <60 years (HR: 1.67; 95%CI:.31–9.11). However, in the subgroup of patients with infarction in anterior circulation, there was no significant interaction, neither in ischemic insular damage and sex (*p* = .3000) nor in ischemic insular damage and age (*p* = .9946).

## 4 DISCUSSION

We found a significant association between ischemic insular damage and the occurrence of SU in patients with AIS. The insular damage, especially the right‐side insular damage, indicated a higher risk of SU in hospitalization. Additionally, this association might be more significant in patients older than 60 years.

In recent years, there has been debate surrounding the use of acid suppression therapy for patients with AIS (Krag et al., 2015). Critically ill patients are at a higher risk of developing gastric mucosal damage, but not all cases progress to clinically significant gastrointestinal bleeding. Acid‐suppressing drugs are widely used in clinical practice due to their safety and accessibility, but the potential subtle risks associated with them have received less attention. These drugs reduce the risk of gastrointestinal bleeding but increase the risk of hospital‐acquired pneumonia (Herzig et al., 2009; MacLaren et al., 2014) and, in some cases, may even raise mortality in critically ill patients (Wang et al., 2020). Nonetheless, there is currently no universally accepted guidance for prophylactic acid suppression therapy in AIS patients, making it important to identify risk factors associated with SU development, especially in AIS cases. Thus, our present study primarily focuses on determining the association between insular damage and the occurrence of SUs during hospitalization.

The pathogenesis of SUs remains incompletely understood, but research suggests that it is a multifactorial phenomenon. One possible explanation for the development of SU is an imbalance between protective and harmful factors in the gastrointestinal mucosa under stress conditions. The insula, a crucial brain region, plays a central role in integrating sensory, interoceptive, emotional, and autonomic processing (Cauda et al., 2012; Gogolla, 2017). Lesions in the insular cortex, both in anterior and posterior regions, have been linked to the occurrence of cardiac arrhythmias. These two major subcomponents of the insula are associated with distinct functions: The posterior insula integrates interoceptive, perceptual, and emotional processes and is functionally connected to sensory cortices, whereas the anterior insula domain mediates cognitive processing and salience and is functionally connected to the anterior cingulate cortex and temporal cortices. Damage to either region can lead to significant autonomic dysfunction and cardiac arrhythmias, with right‐hemisphere insular damage, in particular, being associated with autonomic dysfunction and cardiac arrhythmias (Tomeo et al., 2022). The extent of dysfunction following insular cortex lesions is evidenced by the increased rates of sudden death and consequent increased cardiac morbidity and mortality (Lin et al., 2021). In cases of right hemisphere insular damage, patients often display elevated serum cardiac troponin T levels, indicating myocardial injury and leading to functional impairment (Ay et al., 2006; Gogolla, 2017). In severe cases, this can progress to heart failure (Hermanns et al., 2022), resulting in a reduced ejection fraction, a measure of the heart's pumping ability (Li et al., 2019; Winder et al., 2023). A decreased ejection fraction indicates inefficient heart pumping, leading to reduced blood flow to the gastrointestinal mucosa. The GI mucosa relies heavily on adequate blood flow for its protection and function, with protective factors including epithelial cell renewal in the mucus‐bicarbonate layer, sufficient blood supply, and the presence of protective molecules like prostaglandins. When blood flow decreases, these protective mechanisms may be compromised, rendering the GI mucosa susceptible to harmful factors, such as gastric acid, pepsin, and bile (Cook & Guyatt, 2018; Saeed et al., 2022).

The regulation of gastroduodenal bicarbonate secretion is controlled by autonomic neural pathways. Parasympathetic vagal nerves promote bicarbonate secretion, whereas sympathetic splanchnic nerves exert inhibitory control. When insular infarction occurs, it can induce sympathetic hyperactivity, leading to the release of catecholamines, such as epinephrine, norepinephrine, and dopamine, which increases sympathetic activity and disrupts the delicate balance of bicarbonate secretion. Consequently, there is a decrease in gastroduodenal bicarbonate secretion, impairing the buffering capacity of the gastric and duodenal mucosa (Cai et al., 2022; Fändriks & Jönson, 1990). Therefore, the activation of the sympathetic nervous system can also delay gastric emptying and relaxation of the pylorus, which creates a necessary pressure gradient for retrograde movement of refluxed material from the gastric antrum to the duodenum. Additionally, it can directly damage the gastric mucosa and promote the development of Sus, and this imbalance between protective and destructive factors can lead to the development of SUs.

## 5 CONCLUSIONS

Our study highlights a significant association between ischemic insular damage and SUs, with this relationship remaining statistically significant even after adjusting for various confounding factors. Furthermore, our findings indicate that both left and right insular damage are independently linked to the occurrence of SUs. Insular infarctions, particularly those affecting the right insular region, can contribute to the development of SUs through two distinct mechanisms: indirectly, by impairing cardiac function, and directly, by affecting sympathetic nerve activity. This influence disrupts gastrointestinal bicarbonate secretion, reduces gastrointestinal blood flow, delays gastric emptying, and causes pyloric relaxation, ultimately compromising protective factors while increasing destructive elements. For patients with AIS and concurrent insular damage, the presence of insular damage emerges as a crucial predictive factor for the development of SUs during hospitalization. In these cases, the consideration of proton pump inhibitors as a preventive measure against SUs could be warranted. However, the decision to implement SU prevention during hospitalization is intricate and individualized, necessitating a comprehensive assessment of each patient's individualized risk factors, current clinical conditions, and a balanced evaluation of the potential benefits and risks associated with prophylactic therapy. Thus, a thorough evaluation of these multifaceted factors is needed for the effective management of AIS patients and for minimizing the likelihood of adverse outcomes.

## AUTHOR CONTRIBUTIONS

**Peng Ding**: Writing—original draft; writing—review and editing; software; investigation; formal analysis; data curation; methodology; visualization; validation; conceptualization; supervision. **Guojuan Chen**: Conceptualization; writing—original draft; writing—review and editing; methodology; software; data curation; investigation; validation; formal analysis; supervision; visualization. **Yuling Yang**: Conceptualization; methodology; writing—review and editing. **Tong Zhang**: Writing—review and editing; conceptualization; data curation. **Wenxia Li**: Conceptualization; writing—review and editing; investigation; software; data curation. **Liqin Yang**: Software; writing—review and editing; conceptualization; investigation; data curation. **Xueqing Liu**: Data curation; investigation; conceptualization; writing—review and editing; software. **Delin Yu**: Data curation; methodology. **Wei Yue**: Writing—original draft; writing—review and editing; funding acquisition; resources; project administration; investigation; supervision.

## CONFLICT OF INTEREST STATEMENT

On behalf of all authors, the corresponding author states that there are no conflicts of interest.

### PEER REVIEW

The peer review history for this article is available at https://publons.com/publon/10.1002/brb3.3529.

## CLINICAL TRIAL REGISTRATION

The SPARK (Effect of Cardiac Function on Short‐Term Functional Prognosis in Patients with Acute Ischemic Stroke) study was designed to investigate the relationship of cardiac function with functional outcomes in patients of AIS (https://www.chictr.org.cn/; registration number: ChiCTR2300067696).

## CONSENT FOR PUBLICATION

In the case of our manuscript, we have obtained consent for publication from all individuals whose data, images, or other personal information are included in this article. We have used our institutional consent form for this purpose. We understand that these consent records are confidential but may be requested by the journal at any stage, including after publication.

## Supporting information


**Table S1** Baseline characteristics of patients with left and right insular damages (*n* = 1037).

## Data Availability

The datasets generated and analyzed during the current study will be submitted to the journal as part of the manuscript submission.

## References

[brb33529-bib-0001] Aho, K. , Harmsen, P. , Hatano, S. , Marquardsen, J. , Smirnov, V. E. , & Strasser, T. (1980). Cerebrovascular disease in the community: Results of a WHO collaborative study. Bulletin of the World Health Organization, 58, 113–130. 6966542 PMC2395897

[brb33529-bib-0002] Alhazzani, W. , Alshamsi, F. , Belley‐Cote, E. , Heels‐Ansdell, D. , Brignardello‐Petersen, R. , Alquraini, M. , Perner, A. , Møller, M. H. , Krag, M. , Almenawer, S. , Rochwerg, B. , Dionne, J. , Jaeschke, R. , Alshahrani, M. , Deane, A. , Perri, D. , Thebane, L. , Al‐Omari, A. , Finfer, S. , … Guyatt, G. (2018). Efficacy and safety of stress ulcer prophylaxis in critically ill patients: A network meta‐analysis of randomized trials. Intensive Care Medicine, 44, 1–11. 10.1007/s00134-017-5005-8 PMC5770505 29199388

[brb33529-bib-0003] Ay, H. , Koroshetz, W. J. , Benner, T. , Vangel, M. G. , Melinosky, C. , Arsava, E. M. , Ayata, C. , Zhu, M. , Schwamm, L. H. , & Sorensen, A. G. (2006). Neuroanatomic correlates of stroke‐related myocardial injury. Neurology, 66, 1325–1329. 10.1212/01.wnl.0000206077.13705.6d 16525122

[brb33529-bib-0004] Braga, M. , Gianotti, L. , Gentilini, O. , Parisi, V. , Salis, C. , & Di Carlo, V. (2001). Early postoperative enteral nutrition improves gut oxygenation and reduces costs compared with total parenteral nutrition. Critical Care Medicine, 29, 242–248. 10.1097/00003246-200102000-00003 11246300

[brb33529-bib-0005] Cai, Y. , Lu, X. , Cheng, X. , Lv, Q. , Xu, G. , & Liu, X. (2022). Increased renal dysfunction, apoptosis, and fibrogenesis through sympathetic hyperactivity after focal cerebral infarction. Translational Stroke Research, 13, 641–651. 10.1007/s12975-021-00900-w 33713029

[brb33529-bib-0006] Cauda, F. , Costa, T. , Torta, D. M. E. , Sacco, K. , D'Agata, F. , Duca, S. , Geminiani, G. , Fox, P. T. , & Vercelli, A. (2012). Meta‐analytic clustering of the insular cortex: Characterizing the meta‐analytic connectivity of the insula when involved in active tasks. Neuroimage, 62, 343–355. 10.1016/j.neuroimage.2012.04.012 22521480 PMC4782788

[brb33529-bib-0007] Cook, D. , & Guyatt, G. (2018). Prophylaxis against upper gastrointestinal bleeding in hospitalized patients. New England Journal of Medicine, 378, 2506–2516. 10.1056/NEJMra1605507 29949497

[brb33529-bib-0008] Ephgrave, K. S. , Kleiman‐Wexler, R. L. , & Adair, C. G. (1990). Enteral nutrients prevent stress ulceration and increase intragastric volume. Critical Care Medicine, 18, 621–624. 10.1097/00003246-199006000-00009 2111755

[brb33529-bib-0009] Fändriks, L. , & Jönson, C. (1990). Vagal and sympathetic control of gastric and duodenal bicarbonate secretion. Journal of Internal Medicine Supplement, 732, 103–107. 10.1111/j.1365-2796.1990.tb01480.x 2200412

[brb33529-bib-0010] GBD 2016 Stroke Collaborators . (2019). Global, regional, and national burden of stroke, 1990–2016: A systematic analysis for the Global Burden of Disease Study 2016. Lancet Neurology, 18, 439–458. 10.1016/S1474-4422(19)30034-1 30871944 PMC6494974

[brb33529-bib-0011] Gogolla, N. (2017). The insular cortex. Current Biology, 27, R580–R586. 10.1016/j.cub.2017.05.010 28633023

[brb33529-bib-0012] Guilbert, J. , Bounous, G. , & Gurd, F. N. (1969). Role of intestinal chyme in the pathogenesis of gastric ulceration following experimental hemorrhagic shock. Journal of Trauma, 9, 723–743. 10.1097/00005373-196908000-00010 5798243

[brb33529-bib-0013] Hermanns, N. , Wroblewski, V. , Bascuñana, P. , Wolf, B. , Polyak, A. , Ross, T. L. , Bengel, F. M. , & Thackeray, J. T. (2022). Molecular imaging of the brain‐heart axis provides insights into cardiac dysfunction after cerebral ischemia. Basic Research in Cardiology, 117, 52. 10.1007/s00395-022-00961-4 36279013 PMC9592646

[brb33529-bib-0014] Herzig, S. J. , Howell, M. D. , Ngo, L. H. , & Marcantonio, E. R. (2009). Acid‐suppressive medication use and the risk for hospital‐acquired pneumonia. JAMA, 301, 2120–2128. 10.1001/jama.2009.722 19470989

[brb33529-bib-0015] Kazamias, P. , Kotzampassi, K. , Koufogiannis, D. , & Eleftheriadis, E. (1998). Influence of enteral nutrition‐induced splanchnic hyperemia on the septic origin of splanchnic ischemia. World Journal of Surgery, 22, 6–11. 10.1007/s002689900341 9465754

[brb33529-bib-0016] Krag, M. , Perner, A. , Wetterslev, J. , Wise, M. P. , Borthwick, M. , Bendel, S. , McArthur, C. , Cook, D. , Nielsen, N. , Pelosi, P. , Keus, F. , Guttormsen, A. B. , Moller, A. D. , Møller, M. H. , & SUP‐ICU co‐authors . (2015). Prevalence and outcome of gastrointestinal bleeding and use of acid suppressants in acutely ill adult intensive care patients. Intensive Care Medicine, 41, 833–845. 10.1007/s00134-015-3725-1 25860444

[brb33529-bib-0017] Li, Y. , Fitzgibbons, T. P. , McManus, D. D. , Goddeau, R. P. , Silver, B. , & Henninger, N. (2019). Left ventricular ejection fraction and clinically defined heart failure to predict 90‐day functional outcome after ischemic stroke. Journal of Stroke and Cerebrovascular Diseases, 28, 371–380. 10.1016/j.jstrokecerebrovasdis.2018.10.002 30396839 PMC6320316

[brb33529-bib-0018] Lin, H.‐B. , Li, F.‐X. , Zhang, J.‐Y. , You, Z.‐J. , Xu, S.‐Y. , Liang, W.‐B. , & Zhang, H.‐F. (2021). Cerebral‐cardiac syndrome and diabetes: Cardiac damage after ischemic stroke in diabetic state. Frontiers in Immunology, 12, 737170. 10.3389/fimmu.2021.737170 34512671 PMC8430028

[brb33529-bib-0019] MacLaren, R. , Reynolds, P. M. , & Allen, R. R. (2014). Histamine‐2 receptor antagonists vs proton pump inhibitors on gastrointestinal tract hemorrhage and infectious complications in the intensive care unit. JAMA Internal Medicine, 174, 564–574. 10.1001/jamainternmed.2013.14673 24535015

[brb33529-bib-0020] Saeed, M. , Bass, S. , & Chaisson, N. F. (2022). Which ICU patients need stress ulcer prophylaxis? Cleveland Clinic Journal of Medicine, 89, 363–367. 10.3949/ccjm.89a.21085 35777844

[brb33529-bib-0021] Tomeo, R. A. , Gomes‐de‐Souza, L. , Benini, R. , Reis‐Silva, L. L. , & Crestani, C. C. (2022). Site‐specific regulation of stress responses along the rostrocaudal axis of the insular cortex in rats. Frontiers in Neuroscience, 16, 878927. 10.3389/fnins.2022.878927 35620667 PMC9127339

[brb33529-bib-0022] Wang, Y. , Ge, L. , Ye, Z. , Siemieniuk, R. A. , Reintam Blaser, A. , Wang, X. , Perner, A. , Møller, M. H. , Alhazzani, W. , Cook, D. , & Guyatt, G. H. (2020). Efficacy and safety of gastrointestinal bleeding prophylaxis in critically ill patients: An updated systematic review and network meta‐analysis of randomized trials. Intensive Care Medicine, 46, 1987–2000. 10.1007/s00134-020-06209-w 32833040

[brb33529-bib-0023] Winder, K. , Villegas Millar, C. , Siedler, G. , Knott, M. , Dörfler, A. , Engel, A. , Achenbach, S. , Hilz, M. J. , Kallmünzer, B. , Schwab, S. , Seifert, F. , & Fröhlich, K. (2023). Acute right insular ischaemic lesions and poststroke left ventricular dysfunction. Stroke and Vascular Neurology, 8(4), 301–306. 10.1136/svn-2022-001724 36653066 PMC10512080

[brb33529-bib-0024] Ye, Z. , Reintam Blaser, A. , Lytvyn, L. , Wang, Y. , Guyatt, G. H. , Mikita, J. S. , Roberts, J. , Agoritsas, T. , Bertschy, S. , Boroli, F. , Camsooksai, J. , Du, B. , Heen, A. F. , Lu, J. , Mella, J. M. , Vandvik, P. O. , Wise, R. , Zheng, Y. , Liu, L. , & Siemieniuk, R. A. C. (2020). Gastrointestinal bleeding prophylaxis for critically ill patients: A clinical practice guideline. BMJ, 368, l6722. 10.1136/bmj.l6722 31907223

