# Supplementary material for: Ischemic insular damage and stress ulcer in patients of acute ischemic stroke
Source: Brain Behav. 2024 May 15;14(5):e3529. doi: 10.1002/brb3.3529 (PMC11095302; doi:10.1002/brb3.3529)
Supplement: Supplementary file 1 — Table S1 Baseline characteristics of patients with left and right insular damages (n = 1037). [file BRB3-14-e3529-s001.docx]

**Supplemental Table 1**: Baseline Characteristics of Patients with Left Insular Damage and Right Insular Damage (n=1037).

| Variables | No Insular Damage  (n=878) | Left Insular Damage  (n=80) | Right Insular Damage  (n=79) | P value |
| --- | --- | --- | --- | --- |
| Stress Ulcer, n | 25 (2.8%) | 20 (25.0%) | 24 (30.4%) | <0.0001 |
| Men, n | 642 (73.1%) | 59 (73.8%) | 59 (74.7%) | 0.9513 |
| Age, mean (SD), years | 63.33 (±11.50) | 68.14 (±13.10) | 66.34 (±12.28) | 0.0004 |
| Admission NIHSS score, median (IQR) | 5 (2-8) | 12 (6.25-17) | 12 (8-17) | <0.0001 |
| Systolic Blood Pressure, mean (SD), mmHg | 149.58 (±23.58) | 155.69 (±28.07) | 157.58 (±24.15) | 0.0027 |
| Diastolic Blood Pressure, mean (SD), mmHg | 86.90 (±14.27) | 88.21 (±14.86) | 87.38 (±14.34) | 0.7192 |
| Admission Heart Rate, mean (SD), bmp | 72.34 (±13.16) | 75.33 (±17.16) | 77.86 (17.44) | 0.0010 |
| Hypertension, n | 671 (76.4%) | 56 (70.0%) | 57 (72.2%) | 0.3342 |
| Diabetes, n | 314 (35.8%) | 25 (31.3%) | 28 (35.4%) | 0.7214 |
| Coronary Heart Disease, n | 127 (14.5%) | 18 (22.5%) | 25 (31.6%) | <0.0001 |
| Atrial Fibrillation, n | 34 (3.9%) | 14 (17.5%) | 15 (19.0%) | <0.0001 |
| Living habits, n | | | | |
| Current Smoking | 472 (53.8%) | 47 (58.8%) | 43 (54.4%) | 0.6915 |
| Current Drinking | 261 (29.7%) | 28 (35.0%) | 27 (34.2%) | 0.4685 |
| Stoke Subtype by TOAST, n | | | | <0.0001 |
| Large-Artery Atherosclerosis | 399 (45.4%) | 57 (71.3%) | 52 (65.8%) |  |
| Cardioembolic | 44 (5.0%) | 17 (21.3%) | 18 (22.8%) |  |
| Small-Artery Occlusion | 232 (26.4%) | 1 (1.3%) | 1 (1.3%) |  |
| Other Etiology | 18 (2.1%) | 0 (0.0%) | 1 (1.3%) |  |
| Undetermined | 185 (21.1%) | 5 (6.3%) | 7 (8.9%) |  |
| Massive Cerebral Infarction, n | 6 (0.7%) | 29 (36.3%) | 40 (50.6%) | <0.0001 |
| Intravenous Thrombolysis, n | 57 (6.5%) | 3 (3.8%) | 7 (8.9%) | 0.4217 |
| Endovascular Treatment, n | 3 (0.3%) | 7 (8.8%) | 5 (6.3%) | <0.0001 |
| Anticoagulant Therapy, n | 157 (17.9%) | 23 (28.8%) | 20 (25.3%) | 0.0228 |

HR=Hazard Ratio; CI=Confidence Interval; NIHSS=National Institutes of Health Stroke Scale; TOAST= Trial of ORG 10172 in Acute Stroke Treatment.
